# A Rare Presentation of Retroperitoneal Hemorrhage Following a Spontaneous Vaginal Delivery

**DOI:** 10.7759/cureus.85442

**Published:** 2025-06-05

**Authors:** Vishwantha Rodrigo, Tzu-Yi Chuang

**Affiliations:** 1 General Surgery, Gold Coast Health Service, Gold Coast, AUS

**Keywords:** angioembolization, conservative vs. surgical management, ovarian artery aneurysm, postpartum hemorrhage, retroperitoneal hematoma

## Abstract

Spontaneous retroperitoneal hemorrhage is a rare but potentially fatal condition characterized by bleeding into the retroperitoneal space, located behind the abdominal cavity. In postpartum women, case reports are the primary source of information regarding this rare presentation, which typically arises from uterine artery lacerations, uterine rupture, extension of vaginal hematomas, or, in extremely rare instances, the rupture of ovarian artery aneurysms. Diagnosing a retroperitoneal hemorrhage can be particularly challenging due to its concealed location and the nonspecific nature of its symptoms, often leading to delayed recognition. The management of such bleeds presents additional difficulties, given that surgical access to the retroperitoneal space is complex, and the bleeding may spontaneously tamponade over time. Furthermore, current knowledge of retroperitoneal hemorrhage management is largely based on cases secondary to trauma, which are more common. This knowledge may not always be directly applicable to postpartum spontaneous hemorrhages, making the management of this condition even more complex. As such, it is crucial to document and report these cases to enhance our understanding of postpartum spontaneous retroperitoneal hemorrhages. By doing so, we aim to improve early detection and refine management strategies, ultimately reducing maternal morbidity and mortality associated with this condition. This case describes a 32-year-old woman who presented two days after a spontaneous vaginal birth with a large retroperitoneal bleed secondary to a ruptured ovarian artery aneurysm. She arrived at a rural emergency department in hemorrhagic shock, with a CT scan confirming retroperitoneal bleeding. The patient was urgently transferred to a tertiary center, where exploratory laparotomy was considered. However, she was successfully managed with gelfoam angioembolization and closely monitored with serial imaging.

## Introduction

Retroperitoneal hematoma is characterized by acute or subacute bleeding from retroperitoneal structures, typically following trauma, surgery, or underlying conditions [1-3]. It is exceedingly rare for a retroperitoneal hematoma to occur spontaneously, especially in postpartum women [2]. While postpartum hemorrhage is a well-known complication, with bleeding typically occurring vaginally or intra-abdominally (particularly post-cesarean section), a retroperitoneal bleed in a postpartum patient presenting in shock is rarely suspected. This is because it may present with minimal clinical signs, such as pain, until significant hemorrhage has occurred. Only a few case reports describe spontaneous retroperitoneal hemorrhage in postpartum women, with most identifying the source as a ruptured uterine artery, uterine rupture, or extension of a vaginal hematoma [2]. This case presents an even rarer occurrence, i.e., a postpartum retroperitoneal bleed due to a ruptured ovarian artery aneurysm.

The ovarian arteries are paired vessels arising from the anterolateral aspects of the abdominal aorta at around the level of vertebrae L2/L3. They run inferolateral in the retroperitoneum, continuing between the layers of the suspensory ligament and into the ovaries [4]. Rupture of an ovarian artery aneurysm causing a retroperitoneal bleed is extremely rare, with fewer than 25 cases reported in the English-language literature [5]. The prevalence of ovarian aneurysms is unknown, as they are usually asymptomatic, suggesting that the condition may be underdiagnosed and underreported. Various theories about ovarian artery aneurysm development exist, and they are usually reported in the peripartum period or associated with uterine fibroids and multiparity [5-7].

Evidence indicates that over half of ruptured ovarian artery aneurysms in women aged under 40 years are secondary to pregnancy-related changes. It is theorized that the hormonal and hemodynamic changes during pregnancy lead to the formation or progression of aneurysms. These changes are thought to result from the increased pressure exerted by the growing uterus on the aorta, which causes structural alterations in the arteries. Additionally, increased perfusion of the utero-ovarian arteries during pregnancy may promote aneurysm development. During the postpartum period, branches of the ovarian artery typically undergo involution, but incomplete involution may result in the formation of aneurysms or pseudoaneurysms in future pregnancies [7]. Multiparity, uterine fibroids, hypertension, and vigorous uterine massage have all been implicated as risk factors [8].

Due to the anatomy of the ovarian artery, its rupture can cause bleeding into the retroperitoneal space, which is confined but difficult to access surgically. Historically, life-saving treatment for a ruptured ovarian artery involved surgery, with ligation of the artery both proximal and distal to the rupture. However, more recent case reports describe the successful use of interventional radiology (IR)-guided angioembolization or coiling to manage these aneurysms [6].

In this case, we report a postpartum patient who presented in hemorrhagic shock due to spontaneous retroperitoneal bleeding secondary to a ruptured ovarian artery aneurysm. She was stabilized with rotational thromboelastometry (ROTEM)-guided transfusion, and while surgery was considered, the bleed likely self-tamponaded. The patient then underwent gelfoam angioembolization to prevent rebleeding, and she was successfully managed without the need for surgery. This case emphasizes the importance of considering rare vascular causes of postpartum hemorrhage, such as ovarian artery aneurysm rupture, and highlights the role of IR in managing these patients.

## Case presentation

**Figure 1:**
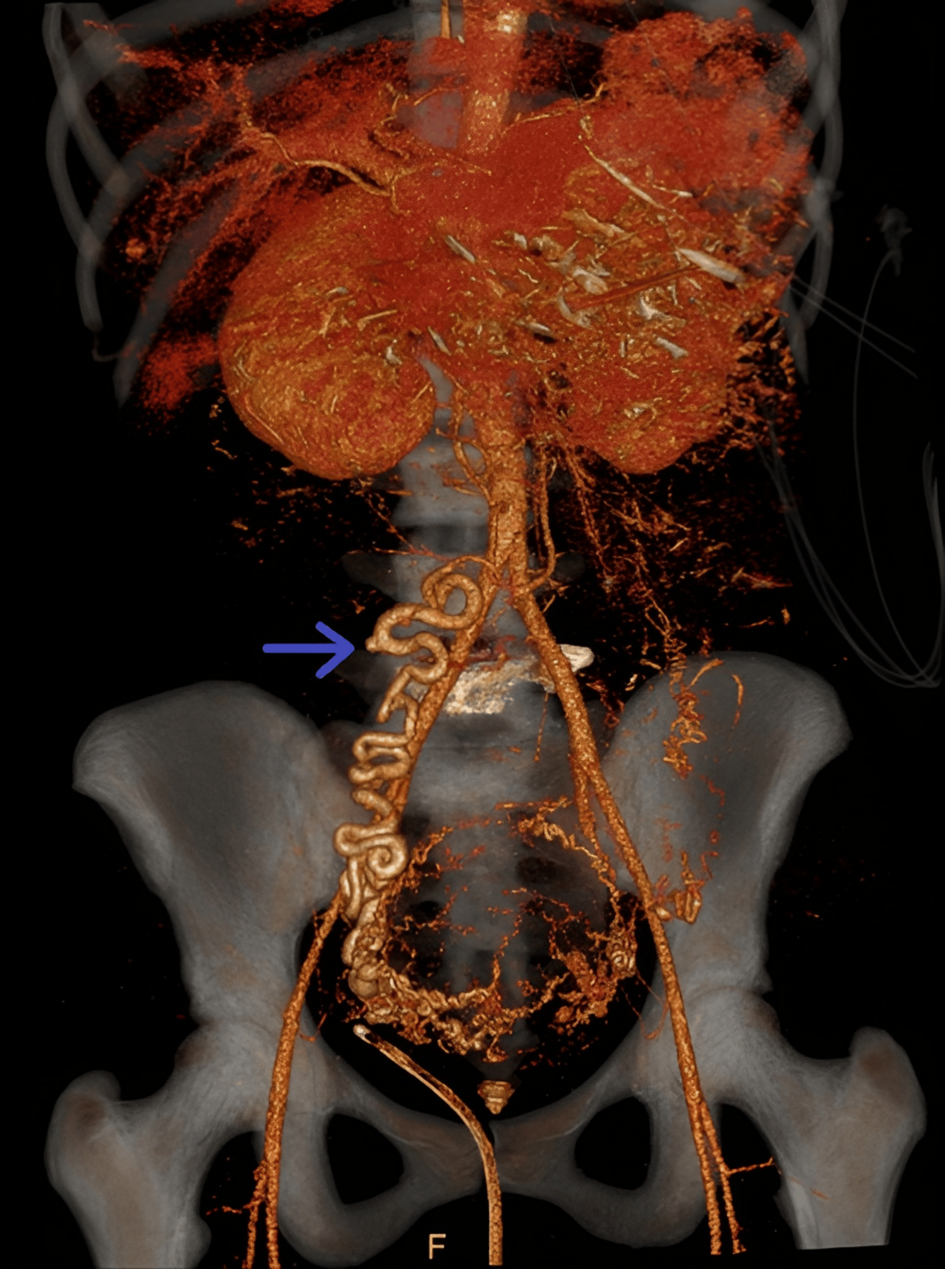
Coronal view of a three-dimensional reconstruction of a CT angiogram scan of the abdomen, with the blue arrow demonstrating the aberrant artery.

**Figure 2:**
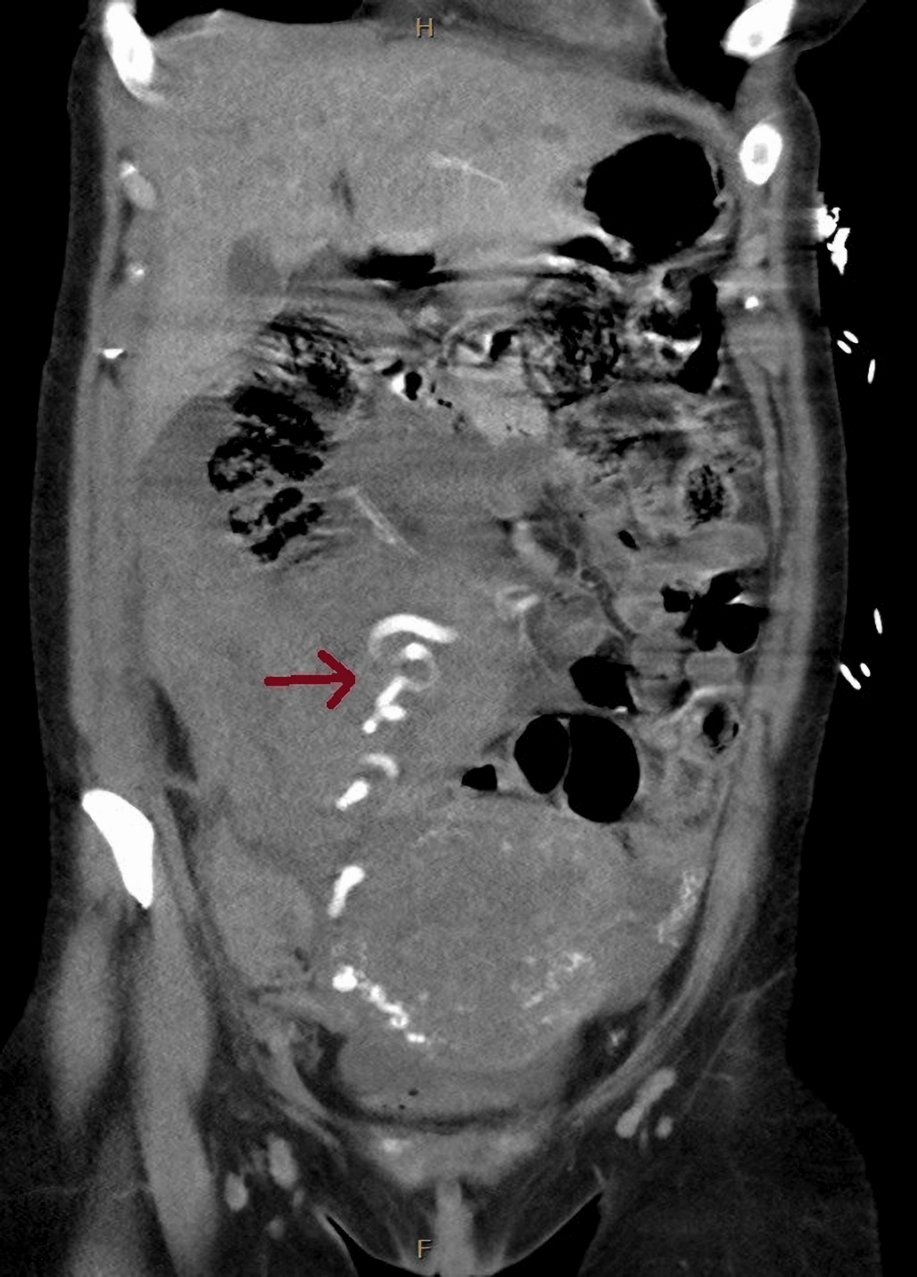
Coronal view of the initial CT angiogram, with the red arrow showing the aberrant artery running through the hematoma.

**Figure 3:**
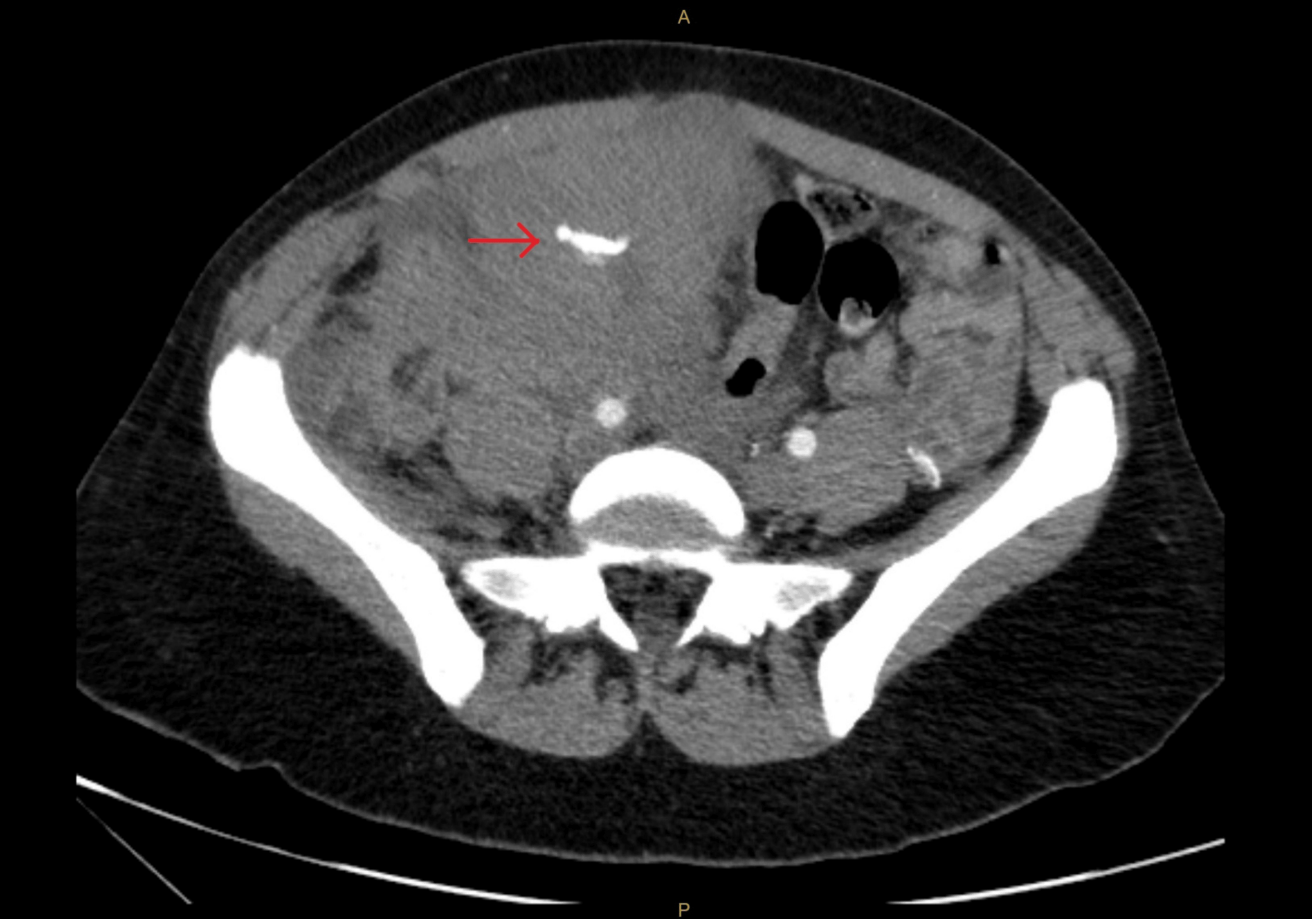
Transverse view of the initial CT angiogram, with the red arrow showing the aberrant artery running through the hematoma.

A 32-year-old female (G6P5) presented to a regional emergency department in hemorrhagic shock two days after an uncomplicated spontaneous vaginal delivery. She developed gradual-onset lower abdominal pain, and, upon arrival, was found to be hypotensive (65/35 mmHg) and tachycardic (heart rate = 110 beats/minute). She was stabilized with two units of packed red blood cells and underwent an urgent CT angiography, which revealed a large volume of hyperdense material in the right hemiabdomen, extending from the pelvis to the right upper quadrant. Initially, the radiology team suspected hemoperitoneum with a large abnormal vessel. However, serial imaging revealed the presence of a large retroperitoneal hematoma, with the abnormal vessel later identified as an ovarian artery aneurysm (Figures 1-3).

Given the findings, the decision was made to transfer the patient to a tertiary center where general surgery and IR services were available. During transport, the patient’s condition deteriorated further into a shocked state, with a blood pressure drop (lowest blood pressure = 60 mmHg), hemoglobin of 60 g/L, and tachycardia (heart rate = 150 beats/minute). She received multiple transfusions (five units of blood, including two units at the Grafton Base Hospital), 1 L of crystalloid, 1 g of tranexamic acid (TXA), calcium, and active warming.

**Figure 4:**
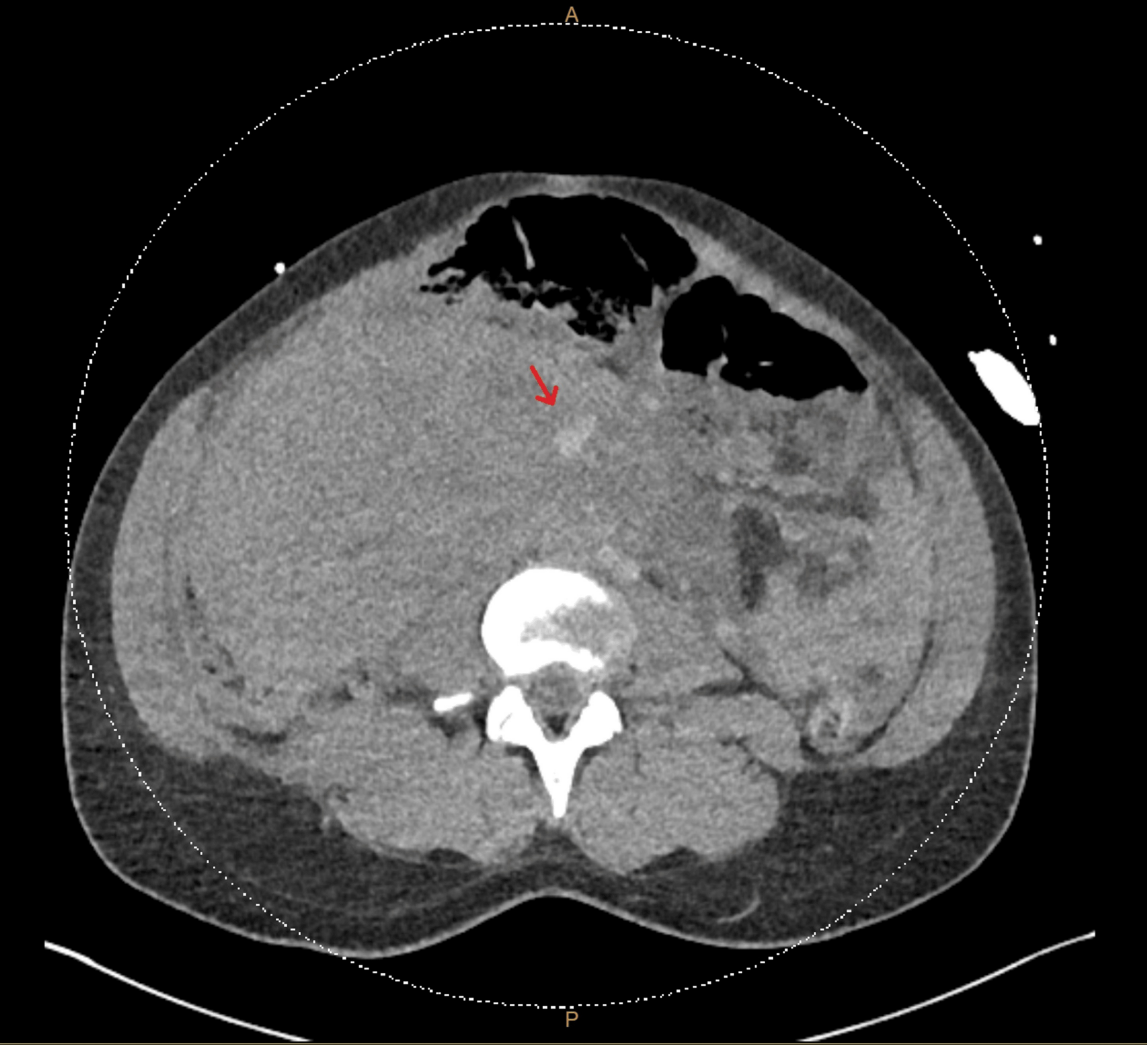
Transverse view of the repeat CT angiogram on arrival to the tertiary hospital, with the red arrow showing contrast extravasation on delayed-phase imaging.

On arrival at the Gold Coast University Hospital, a repeat CT angiogram showed an increase in the size of the acute right retroperitoneal hemorrhage, with multiple small blushes on delayed-phase imaging (Figure 4). Hematoma was also noted within the pelvis, along with some free fluid in the intraperitoneal cavity. The aberrant vessel seen at the regional hospital was not well visualized, possibly due to the imaging phase. Consequently, the use of IR-guided embolization was uncertain, and laparotomy was considered.

The patient received further transfusions guided by ROTEM, including seven units of packed red blood cells, two units of fresh frozen plasma, calcium gluconate (total 6.6 mmol), 1 g TXA, 3 g fibrinogen concentrate, and 1 L of crystalloid. Upon review by the general surgery team, the patient remained hemodynamically stable. Examination revealed a distended abdomen with a very tender, firm mass palpable in the right flank, while the left side of her abdomen was soft. Given her stable condition and the likelihood of self-tamponade of the retroperitoneal bleed, the decision was made to observe her in the intensive care unit (ICU).

Over the weekend, the patient remained stable while awaiting a multidisciplinary team (MDT) discussion. On Monday, senior staff from the IR, obstetrics and gynecology, and general surgery teams reviewed the case. Consensus was that the aberrant vessel represented an aneurysmal right ovarian artery, raising concerns about the potential for rebleeding, particularly as the patient lived in a rural area far from tertiary care centers. Additionally, the patient had a family history of aneurysms (her father had died from a cerebral aneurysm), and she had completed her family from a fertility perspective. The MDT agreed that selective gelfoam angioembolization of the right ovarian artery should be performed as a preventative measure.

The angioembolization procedure was successfully completed without intraoperative complications. The patient was then transferred from the ICU to the general ward. A repeat CT scan following the IR embolization showed a decrease in the size of the retroperitoneal hematoma, and the patient was subsequently discharged home.

## Discussion

Retroperitoneal hematomas are rare, life-threatening complications of childbirth, with few documented cases in the literature. Postpartum hemorrhage is usually managed by obstetricians due to common causes such as uterine atony, retained products of conception, or perineal lacerations [9]. However, retroperitoneal hemorrhage presents a more complex and rarer situation, turning it into a general surgical emergency. Given its rarity and the difficulty in diagnosis and management, retroperitoneal bleeding after childbirth requires high suspicion and often involves input from multiple specialties, including obstetrics, general surgery, and IR. Its unusual nature means general surgeons may rarely encounter such cases, making timely recognition and appropriate intervention critical for patient outcomes [10].

Retroperitoneal hematomas typically arise from injuries to branches of the internal iliac arteries and are rare complications of various obstetric and gynecological procedures. Most reported cases involve uterine artery lacerations, often associated with hysterectomy, uterine rupture, or extensions of paravaginal hematomas. Spontaneous puerperal retroperitoneal hematomas, though even rarer, have been reported, often related to underlying vascular anomalies, such as arterial aneurysms, arteriovenous malformation, and rupture of major pelvic veins, as well as conditions such as eclampsia or blood dyscrasias [11].

The management of retroperitoneal bleeding is particularly challenging due to the anatomical complexity and difficulty in surgically accessing the retroperitoneal space. Retroperitoneal bleeds are more commonly described in trauma literature, where the retroperitoneum is divided into three zones based on anatomical location. Zone 1 includes the midline retroperitoneum (abdominal aorta and vena cava), Zone 2 includes the perinephric space, and Zone 3 encompasses the pelvic retroperitoneum. In trauma cases, Zone 1 bleeds typically require surgical intervention due to the high risk of vascular injury, whereas Zone 2 and Zone 3 bleeds can sometimes be managed conservatively in hemodynamically stable patients, as these areas are more prone to self-tamponade and are harder to access surgically. However, this approach has not been validated for spontaneous bleeds [3].

In this case, the hematoma was primarily located in Zone 2, with extension into Zone 3. Notably, this is a non-traumatic bleed, with a known source, a ruptured ovarian artery aneurysm. Thus, management decisions (surgery, IR, or conservative treatment) must be made on a case-by-case basis, considering the patient’s clinical status and available resources.

The choice of management depends on the severity of the hemorrhage, the patient’s hemodynamic stability, and the availability of resources such as IR. Surgical management aims to achieve hemostasis by accessing the retroperitoneal space to ligate the lacerated vessel. Packing the retroperitoneal space to tamponade the hemorrhage has been reported in trauma cases involving pelvic fractures [3]. However, only case reports describe managing postpartum retroperitoneal bleeds, with most patients requiring laparotomy, extensive dissection, and vessel ligation for hemostasis. Laparotomy, especially in the immediate postpartum period, carries higher risks of mortality, complications, and poor recovery for both mother and baby [2,10].

Recent case reports highlight the success of IR-guided embolization in postpartum retroperitoneal bleeds, both as a curative and preventative measure. IR embolization is a minimally invasive procedure that can control bleeding effectively without the risks associated with surgery [12]. In cases where IR is not available or unsuccessful, surgical intervention may be necessary, where direct visualization and clipping of the bleeding artery is required. In some cases, especially when the patient is stable and the bleeding has self-tamponaded, close observation and monitoring may be appropriate, with readiness for intervention if signs of deterioration or rebleeding occur [10].

This case emphasizes the importance of early multidisciplinary involvement and careful decision-making based on the patient’s hemodynamic status and risk factors. A high level of suspicion is required for retroperitoneal bleeding in postpartum patients, and IR embolization may be considered as a first-line option when available, given its effectiveness and lower risk profile compared to surgery.

## Conclusions

This case illustrates the management of a rare presentation of retroperitoneal bleeding as a postpartum complication and underscores the importance of a multidisciplinary approach in managing complex obstetric emergencies. Early recognition of hemorrhagic shock and the use of advanced imaging techniques, such as CT angiography, were pivotal in diagnosing the source of bleeding. The decision to proceed with selective angioembolization was a tailored and thoughtful intervention, preventing a potentially catastrophic rebleed while considering the patient’s family history and future reproductive needs. Ultimately, the patient’s condition stabilized, and she was discharged without further complications, highlighting the effectiveness of timely intervention in a critically ill obstetric patient.
